# *Aspergillus niger* as a New Endophytic Fungus From *Deverra tortuosa*: Phytochemical Profile, Molecular Docking, Antimicrobial, Antibiofilm, Antioxidant, and Anticancer Activities

**DOI:** 10.1002/mbo3.70430

**Published:** 2026-10-07

**Authors:** Samy Selim, Amr H. Hashem, Amer Morsy Abdelaziz, Mohamed H. Sharaf, Samiah Hamad Al‐Mijalli, Mohammed Aladhadh, Emad M. Abdallah, Mohammed H. Alruhaili, Hattan S. Gattan, Alaa A. Kashmiry, Mohammed Aufy

**Affiliations:** ^1^ Department of Clinical Laboratory Sciences, College of Applied Medical Sciences Jouf University Sakaka Saudi Arabia; ^2^ Botany and Microbiology Department, Faculty of Science Al‐Azhar University Nasr City, Cairo Egypt; ^3^ Department of Biology, College of Sciences Princess Nourah bint Abdulrahman University Riyadh Saudi Arabia; ^4^ Department of Food Science and Human Nutrition, College of Agriculture and Food Qassim University Buraydah Buraydah Saudi Arabia; ^5^ Department of Biology, College of Science Qassim University Buraydah Saudi Arabia; ^6^ Department of Clinical Microbiology and Immunology, Faculty of Medicine King Abdulaziz University Jeddah Saudi Arabia; ^7^ Special Infectious Agents Unit, King Fahad Medical Research Center King Abdulaziz University Jeddah Saudi Arabia; ^8^ Department of Medical Laboratory Sciences, Faculty of Applied Medical Sciences King Abdulaziz University Jeddah Saudi Arabia; ^9^ Department of Biological Sciences, College of science University of Jeddah Jeddah Saudi Arabia; ^10^ Department of Pharmaceutical Sciences, Division of Pharmacology and Toxicology University of Vienna Vienna Austria

**Keywords:** antibiofilm, antimicrobial, antioxidant, *Aspergillus*, bioactive compounds, *Deverra tortuosa*, endophytes

## Abstract

Endophytic fungi are recognized as rich sources of bioactive secondary metabolites with promising pharmaceutical applications. In this study, five endophytic fungal isolates were obtained from *Deverra tortuosa* and evaluated for antimicrobial activity for the first time. Among them, isolate AA1 showed the highest antimicrobial potential and was identified as *Aspergillus niger* (GenBank accession no. PX273401.1) through morphological and molecular analyses. Phytochemical analysis revealed high levels of flavonoids (496.5 µg/mL), tannins (260.7 µg/mL), phenolics (214.93 µg/mL), and alkaloids (26.45 µg/mL). GC–MS profiling detected several bioactive metabolites, with hexadecenoic acid, docosene, octadecanol, isochiapin B, octadecenoic acid, and octacosanol as major constituents. The fungal extract exhibited potent antibacterial activity against *Staphylococcus aureus*, *Klebsiella pneumoniae*, and *Bacillus cereus*, each with a minimum inhibitory concentration (MIC) of 62.5 µg/mL. It also inhibited biofilm formation by 26.9:55.1% at ½ MIC and demonstrated notable antioxidant activity, with half maximal inhibitory concentration (IC_50_) values of 167.6 µg/mL (DPPH) and 137.8 µg/mL (ABTS). Cytotoxicity assays confirmed relatively low toxicity toward normal Wi‐38 and Vero cell lines, while significant antiproliferative activity was observed against MCF‐7 and HepG2 cancer cells, with IC_50_ values of 78.7 and 29.5 µg/mL, respectively, indicating favorable selectivity. Molecular docking supported these biological activities, identifying hexadecenoic acid as the strongest ligand against DNA gyrase subunit B (GyrB) and estrogen receptor alpha (ERα), while isochiapin B also showed strong affinity toward ERα. These findings demonstrate that *A. niger* from *D. tortuosa* is a promising source of multifunctional bioactive metabolites with antimicrobial, antioxidant, and anticancer potential for future pharmaceutical applications.

## Graphical abstract

*Aspergillus niger* AA1 isolated from *Deverra tortuosa* produced a metabolite‐rich extract with antibacterial, antibiofilm, antioxidant, and selective anticancer activities. Gas chromatography–mass spectrometry identified major bioactive compounds, while molecular docking supported their interactions with DNA gyrase B and estrogen receptor alpha, highlighting the extract as a promising source of multifunctional bioactive metabolites.

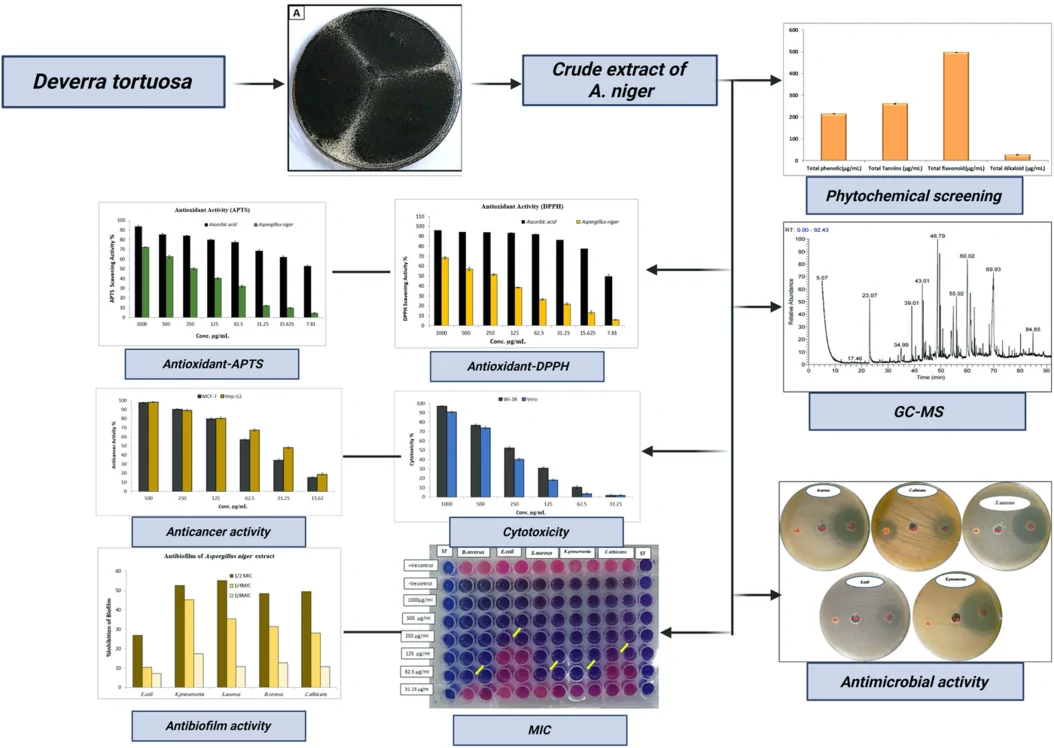

## 1 Introduction

The treatment of infectious diseases is becoming more challenging due to the growing global health emergency posed by multidrug‐resistant microorganisms (MDRMs). *Escherichia coli*, *Klebsiella pneumoniae*, and *Staphylococcus aureus* are among the MDRM that have become resistant to several antibiotics, drastically limiting the range of available treatments (Shehabeldine et al. 2025). Resistance has developed more quickly due to the extensive and often improper use of antibiotics in both agriculture and human medicine (Soliman et al. 2024; Elshamy et al. 2025). Longer hospital stays, higher healthcare expenses, and higher mortality rates are all consequences of MDRM infections (Abdel Fattah et al. 2025). Additionally, the rapid dissemination of resistance genes among bacterial populations made possible by horizontal gene transfer complicates control attempts (Munita and Arias 2016; Khedr et al. 2026). Biofilm formation is one of the virulence strategies that help pathogenic microorganisms survive antimicrobial and environmental stresses. Biofilms are organized microbial communities within an extracellular matrix, and their development and various virulence‐related activities are controlled by quorum sensing (QS) (Choudhury et al. 2026). Thus, microbial adhesion, extracellular matrix production, QS signaling, or established biofilm structures related strategies have emerged as promising for the control of persistent infections. In this context, endophytic microorganisms are valuable sources of diverse bioactive metabolites with antimicrobial, antibiofilm, and QS‐inhibitory activities. However, their practical application requires careful evaluation of biological selectivity, toxicity, ecotoxicological effects, and environmental safety to develop effective and sustainable alternatives against resistant pathogens (Kalia et al. 2023; Elebiary et al. 2026).

Endophytic microorganisms live asymptomatically inside plant tissues and establish symbiotic relationships without appearing to harm the host (Hasan 2026a). The plant kingdom is home to endophytes, which can live in roots, stems, leaves, seeds, and flowers, among other plant parts (Abdelaziz 2025). Endophyte habitats are highly variable and influenced by geographic location, plant species, and environmental conditions. Endophytic diversity and distribution are influenced by soil type, climate, and the plant's physiological state (Compant et al. 2010). Endophytes, which are present in plants that grow in harsh conditions such as deserts or saline soils, frequently have specialized stress‐tolerance traits that increase their hosts' resistance to abiotic stresses (Kamran et al. 2022). Endophytes are a renewable and sustainable source of new bioactive substances (Hasan 2026b). They generate a wide range of bioactive compounds, including phenolics, flavonoids, terpenoids, alkaloids, and peptides (Attia 2025a). Endophytes' capacity to produce these metabolites has significant ramifications for numerous industries. Numerous endophytic compounds are promising candidates for drug development in the pharmaceutical industry because of their strong antimicrobial, anticancer, anti‐inflammatory, and antioxidant properties (Hallmann et al. 1997).

Bacterial endophytes have been reported to produce antimicrobial metabolites, enzymes and other bioactive compounds (Hashem 2023a). Recently, evidence has demonstrated the capacity of bacterial endophytes to inhibit pathogenic bacteria through antimicrobial, antibiofilm, and quorum‐sensing‐linked mechanisms. Conversely, fungal endophytes are of particular interest for therapeutic bioprospecting due to their broad biosynthetic capacity and ability to produce structurally diverse secondary metabolites, including antimicrobial, antioxidant, antiviral, and anticancer compounds. Therefore, both bacterial and fungal endophytes are important producers of bioactive molecules, but fungal endophytes may be especially advantageous for pharmaceutical discovery because of their extensive and chemically diverse secondary metabolite repertoire (Tiwari and Bae 2022). The therapeutic superiority of fungal versus bacterial endophytes cannot be generalized, but is dependent on the endophyte, host plant, metabolites produced and desired application. Fungal endophytes are being explored for novel bioactive metabolites with therapeutic applications (Nath et al. 2026; Prajapati et al. 2025).

*Deverra tortuosa* is an aromatic perennial shrub that is native to the arid and semiarid regions of North Africa and the Middle East, where it is highly adapted to harsh environmental conditions. It thrives in sandy, rocky, and poor soils and is frequently found in steppe and desert environments (Quezal and Santa 1996). Due to its many therapeutic benefits, *D. tortuosa* exploited in conventional medicine. Important biological activities, as antimicrobial, antioxidant, anti‐inflammatory, and insecticidal effects, have been shown by extracts from various plant parts, particularly the aerial parts and essential oils (Attia et al. 2023; Hashem 2023b). Its medicinal efficacy is thought to be attributed to the presence of bioactive compounds like flavonoids, coumarins, and essential oils that are rich in monoterpenes and sesquiterpenes, as demonstrated by phytochemical studies (Al‐Faraj 1992; Batanouny 2000).

The lack of prior studies on the endophytic fungi of *D. tortuosa*, specifically their capacity to produce bioactive compounds with pharmacological applications, represents the research gap this study seeks to address. There are no reports of fungal isolates from *D. tortuosa* or of their metabolites, despite many medicinal plants being known to harbor endophytic fungi with antioxidant and antimicrobial properties. This limits our understanding of their potential as eco‐friendly alternative sources of novel therapeutic agents to combat infections caused by biofilms and multidrug‐resistant pathogens. To assess the antimicrobial, antibiofilm, antioxidant, and anticancer properties of the extract and to correlate these bioactivities with its phytochemical composition and gas chromatography–mass spectrometry (GC–MS)‐derived bioactive compounds, our study set out to isolate and identify fungal endophytes from *D. tortuosa* as novel natural products with potential pharmaceutical applications.

## 2 Material and Methods

### 2.1 Isolation and Identification of Endophytic Fungi

Healthy leaves of *D. tortuosa* (L.) DC were collected and aseptically cut into 5 × 5 mm segments. Three segments were placed per Petri dish in triplicate. The segments were washed twice with sterile distilled water, surface‐sterilized with 70% ethanol for 30 s followed by 4% NaOCl for 3 min, and rinsed three times with sterile distilled water. Sterilization efficiency was confirmed by plating 100 μL of the final rinse water and imprinting randomly selected segments onto potato dextrose agar (PDA) plates; no microbial growth was observed. The sterilized segments were then placed on PDA and incubated at 28°C for 21 days to recover endophytic fungi. Fungal growth was monitored daily and the emerged colonies were purified by repeated hyphal‐tip subculturing and preserved on PDA for further morphological, molecular, and phytochemical. The isolation frequency of the recovered endophytic fungal isolate was 100% (9/9 leaf tissue segments), suggesting successful recovery of the endophyte from all cultured segments (Abdelaziz et al. 2023). The antimicrobial activity of the fungal isolates was examined. On the basis of molecular analysis, growth characteristics, and colony morphology, the most potent fungal strain was determined. Internal Transcribed Spacer (ITS) region was amplified and examined in accordance with established protocols after DNA was extracted for molecular identification (Shehabeldine et al. 2024).

### 2.2 Extraction of *Aspergillus niger* Bioactive Compounds (Preparation of *A. niger* Extract)

*A. niger* was cultured in 500 mL sterile potato dextrose broth (pH 5.6 ± 0.2) in 1 L Erlenmeyer flasks. Each flask was inoculated with 1 × 10^6^ spores/mL and incubated statically at 28°C for 15 days. The culture was filtered through Whatman No. 1 filter paper, and the cell‐free filtrate was extracted with an equal volume of ethyl acetate (1:1, v/v). After vigorous shaking for 10 min and an overnight phase separation at 4°C, the organic phase was collected and evaporated at 40°C, yielding 0.1 g crude ethyl acetate extract (EACE). The extract was stored at 4°C and redissolved in dimethyl sulfoxide (DMSO) to 1000 µg/mL before analyses. DMSO was maintained at ≤ 1% (v/v) in all assays with appropriate solvent controls. Sterile uninoculated potato dextrose broth was extracted similarly as a negative control (Khalil et al. 2021).

### 2.3 Phytochemical Analysis of *A. niger* Extract

The phytochemical analysis of *A. niger* extract was performed according to Shehata et al. (2024). Total flavonoid content was determined using the AlCl_3_ colorimetric method. Briefly, 200 μL of methanolic extract was mixed with 75 μL of 5% NaNO_3_ and incubated for 5 min, followed by the addition of 0.5 mL 5% NaOH and 1.25 mL 5% AlCl_3_. After sonication and 5 min incubation, absorbance was measured at 510 nm. Total phenolic content (TPC) was determined using the Folin–Ciocalteu method. A 500‐μL extract solution was mixed with 2.5 mL FC reagent and 2.5 mL sodium carbonate (75 g/L), incubated at 25°C for 2 h, and measured at 765 nm. Total tannin content was determined using the vanillin–HCl method. Briefly, 400 μL extract was mixed with 3 mL 4% vanillin and 1.5 mL concentrated HCl, incubated at 25°C for 15 min, and measured at 500 nm using a tannic acid standard curve. Alkaloid content was determined colorimetrically. The extract was washed three times with chloroform, adjusted to pH 7 with 0.1 N NaOH, and mixed with phosphate buffer (pH 4.7) and *Bacillus* Calmette–Guérin (BCG) solution. The alkaloid–BCG complex was extracted into chloroform, and absorbance was measured at 470 nm.

### 2.4 GC‐MS of *A. niger* Extract

The volatile and semi‐volatile bioactive constituents of the *A. niger* extract were identified by GC–MS using a Thermo Scientific Trace GC 1310 coupled to an single quadrupole mass spectrometer and equipped with a trace gold‐5MS capillary column (30 m × 0.25 mm inner diameter and 0.25 µm film thickness). The oven temperature was initially set at 50°C, increased to 230°C at 5°C/min and held for 2 min, then raised to 290°C and held for 2 min. Helium was used as the carrier gas at 1 mL/min, with split injection (1:30) and a 1‐µL injection volume. Mass spectra were acquired in EI mode at 70 eV over *m*/*z* 40–1000, with the ion source at 200°C. Compounds were identified by comparing their mass spectra and retention characteristics with NIST 11 and WILEY 09 databases (M. Sharaf 2020).

### 2.5 Antimicrobial Activity of *A. niger* Extract

The antimicrobial activity of *A. niger* extract was evaluated against MDR clinical isolates of *S. aureus, K. pneumoniae, E. coli*, and *Bacillus cereus*, and *Candida albicans* ATCC 10231.

Antimicrobial activity was assessed by the agar well diffusion method using 100 μL extract per 6‐mm well. Piperacillin (100 μg) and fluconazole (25 μg) were used as positive controls for bacteria and yeast, respectively. Bacteria and *C. albicans* were incubated at 37°C for 24 h and 28°C for 48 h, respectively. Inhibition zones were measured in mm (El‐Sayed et al. 2022; M. H. Sharaf et al. 2026; Hsueh et al. 2010). Minimum inhibitory concentrations (MICs) were determined by broth microdilution according to CLSI M07 and M27 guidelines using resazurin (Cockerill 2012; Fothergill 2011). Twofold extract dilutions (7.81–1000 μg/mL) were prepared in Mueller–Hinton broth (MHB) for bacteria and RPMI 1640 for *C. albicans*. Microbial suspensions were adjusted to approximately 5 × 10^5^ CFU/mL and mixed with equal volumes of extract in 96‐well plates. Growth, sterility, solvent, and positive antimicrobial controls were included. After incubation, resazurin was added, and the MIC was defined as the lowest concentration maintaining a blue color, indicating complete growth inhibition.

### 2.6 Effect of the *A. niger* Extract on Biofilm Biomass

The antibiofilm activity of *A. niger* extract was evaluated using a 96‐well microtiter plate method (Fareid et al. 2025). Briefly, 100 μL MHB and 10 μL microbial suspension (OD_620_ = 0.05 ± 0.02) were mixed with 100 μL extract at ½, ¼, and ⅛ MIC. Untreated inoculated wells served as controls. After incubation at 37°C for 48 h, planktonic cells were removed, and attached biofilms were washed, fixed with absolute ethanol (30 min), and stained with 0.1% crystal violet (30 min). After washing and drying, the bound dye was solubilized with 200 μL 33% acetic acid, and absorbance was measured at 630 nm. The inhibitory effect of *A. niger* extract on biofilm formation was expressed as the decrease in optical density (OD) compared with the untreated control, as shown in Equation (1).

(1) $$\text{Biofilm inhibition}\left(\%\right)=1-\frac{{\text{OD}}_{630}\;\text{of cells treated with different concentration of}\;A.\mathrm{niger}\;\text{extract}}{{\text{OD}}_{630}\;\text{of nontreated control}}\times 100.$$

### 2.7 Antioxidant Activity

#### 2.7.1 DPPH Assay

Antioxidant activity was assessed using 0.1 mM 1,1‐diphenyl‐2‐picrylhydrazyl (DPPH) (Khalil et al. 2021). Extract concentrations (7.81–1000 μg/mL; 100 μL) were mixed with 100 μL DPPH and incubated in darkness for 30 min at room temperature. Absorbance was measured at 517 nm, using ascorbic acid as the reference. Appropriate controls and sample blanks were included. The percentage of DPPH radical scavenging activity was calculated from the corrected absorbance values using Equation (2):

(2) $$\text{DPPH scavenging activity}=\frac{\text{Absorbance of control}-\text{Absorbance of}\;A.\mathrm{niger}\;\text{extract}}{\text{Absorbance of control}}\times 100.$$

#### 2.7.2 ABTS Assay

ABTS^·^^+^ was prepared from 7 mM 2,2′‐azino‐bis‐3‐ethylbenzthiazoline‐6‐sulfonic acid (ABTS) and 2.45 mM potassium persulfate and incubated in darkness for 12–16 h. The solution was diluted to OD_734_ = 0.70 ± 0.02. Extract (100 μL; 7.81–1000 μg/mL) was mixed with 100 μL ABTS^·^^+^ and incubated for 6 min. Absorbance was measured at 734 nm, with ascorbic acid as a reference and extract blanks as controls. The percentage of ABTS radical scavenging activity was calculated with the use of corrected absorbance values, according to Equation (3). Half maximal inhibitory concentration (IC_50_) value was calculated from concentration–response curve (Lee et al. 2015).

(3) $$\text{ABTS scavenging activity}=\frac{\text{Absorbance of control}-\text{Absorbance of}\;A.\mathrm{niger}\;\text{extract}}{\text{Absorbance of control}}\times 100.$$

### 2.8 Cytotoxicity and Anticancer Activity

The cytotoxic effects of *A. niger* extract were evaluated against normal WI‐38 and Vero cells and cancerous Hep‐G2 and Michigan Cancer Foundation‐7 (MCF‐7) cells using the MTT assay at 31.25–500 μg/mL (Van de Loosdrecht et al. 1994). Absorbance was measured at approximately 560 nm in triplicate, and cell viability was calculated (Equations 4 and 5). The selectivity index (SI) was determined as the ratio of IC_50_ in normal cells to that in cancer cells (Equation 6).

(4) $$\text{Viability}\%=\frac{{\text{Sample OD}}_{560}}{{\text{Control OD}}_{560}}\times 100,$$

(5) Cytotoxicity%=100−Viability%,

(6) $$\mathrm{SI}=\frac{{\mathrm{IC}}_{50(\mathrm{normal}\;\mathrm{cells})}}{{\mathrm{IC}}_{50(\mathrm{cancer}\;\mathrm{cells})}}.$$

### 2.9 Molecular Docking Experiment

The molecular structures of the selected compounds were constructed and energy minimized before being converted into a suitable file format for molecular docking analysis, following previously reported procedures (El‐Tabakh et al. 2025; Bashar et al. 2024). The three‐dimensional crystallographic structures of DNA gyrase subunit B (GyrB; PDB ID 3G7B) and estrogen receptor alpha (ERα; PDB ID 3ERT) were obtained from the Protein Data Bank (PDB). The crystal waters were removed, and hydrogen atoms were added before the docking simulations to ensure proper protonation and structural integrity of protein structures. The reliability and accuracy of the docking protocol were confirmed by re‐docking the native co‐crystallized ligands into the active sites of their respective target proteins. The obtained binding conformations were then compared with the experimental poses to validate the docking procedure's capability to accurately reproduce the native ligand–protein interactions (Shehata et al. 2023; N. Ahmed et al. 2023).

### 2.10 Statistical Analysis

Experiments were performed in three independent trials with three replicates. Data were analyzed by one‐way ANOVA, and means were compared using least significant difference at *p* ≤ 0.05. CoStat software was used for statistical analysis.

## 3 Results

### 3.1 Isolation and Identification of Endophytic Fungi

Five fungal isolates (AA1–AA5) were isolated from *D. tortuosa* and tested for their activity as antimicrobials. Fungal isolate AA1 was selected as the most potent isolate among all isolates. Fungal isolate AA1 was identified morphologically as *A. niger* where fungal colonies on PDA medium at 25°C appeared white at first, but conidial production quickly turns them black (Figure 1A). The colony's back side is a light‐yellow color (Figure 1B). The hyphae are hyaline and septate. Conidial heads are radiate structures at first, but as they develop, they split into columns. Long, smooth, and hyaline, conidiophores darken progressively as they approach the apex and terminate in a globose vesicle (Figure 1C). Layers of metulae and phialides completely encase the vesicle. Conidia have a rough surface texture, are globose, and range in color from dark brown to black (Figure 1D). Molecular identification confirmed fungal isolate AA1 identified as *A. niger* with a similarity of approximately 98% (Figure 2). Moreover, the sequence was deposited in GenBank with accession no. PX273401.1.

**Figure 1:**
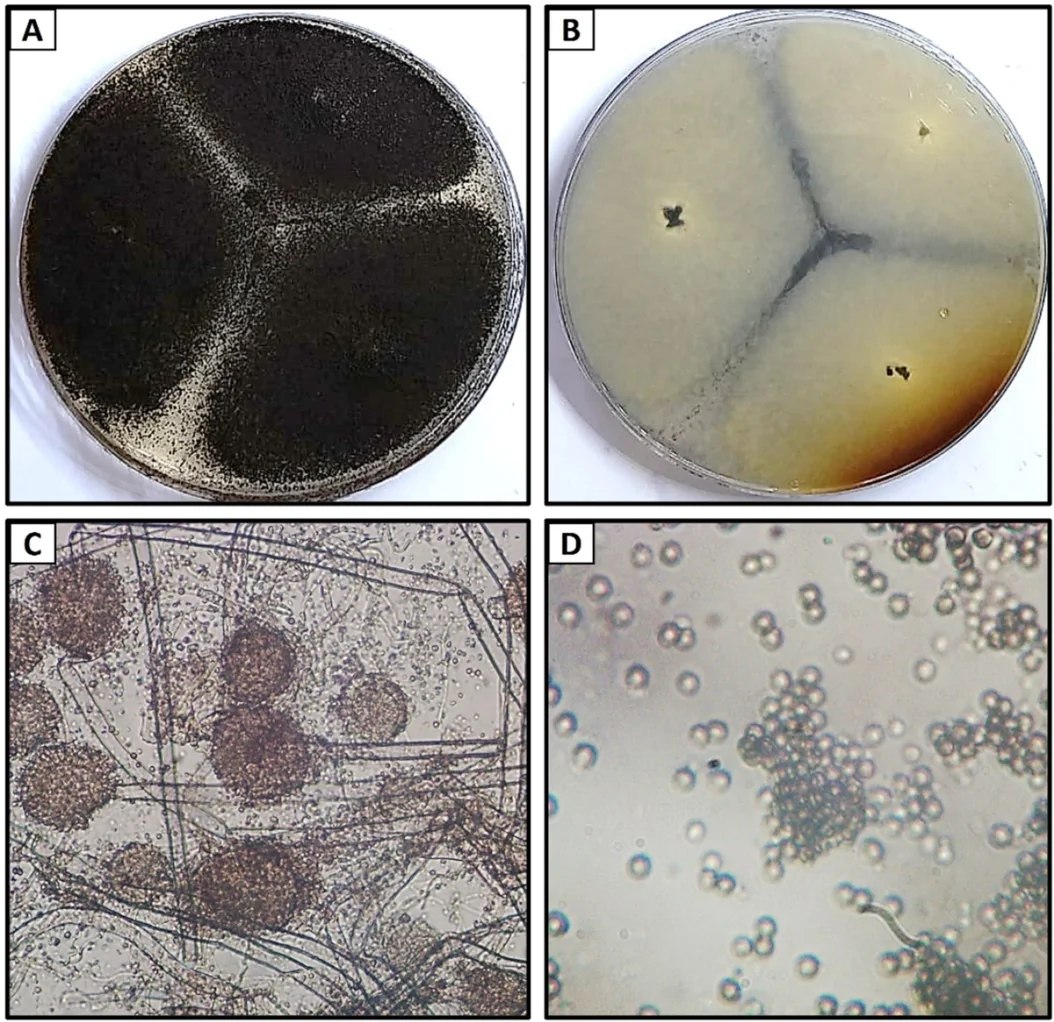
Morphological identification of *Aspergillus niger*: (A) surface, (B) reverse, (C) conidiophore and head, and (D) conidia.

**Figure 2:**
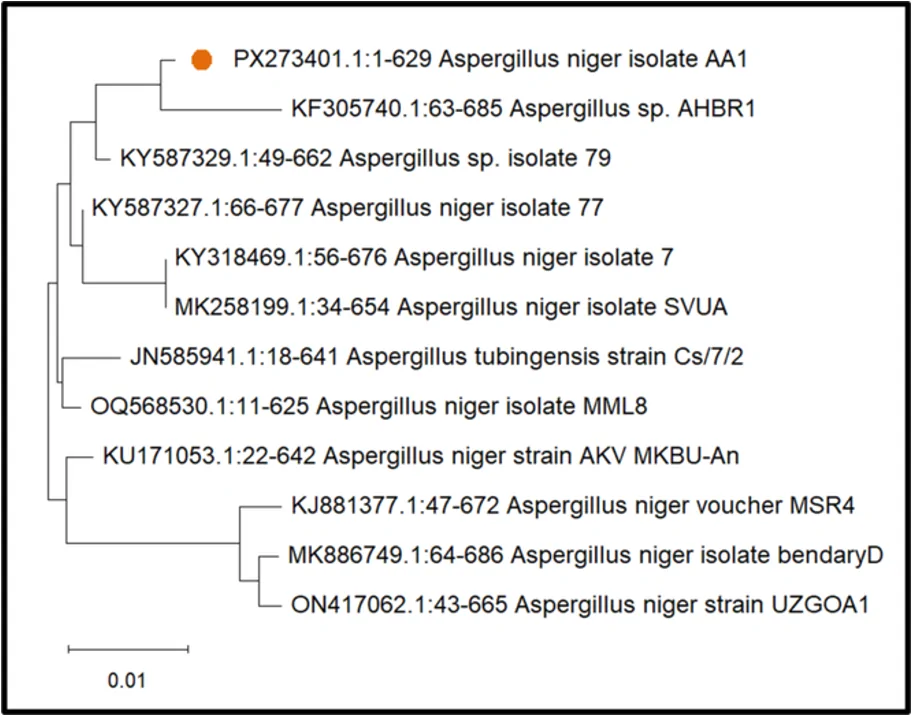
Phylogenetic tree of *Aspergillus niger* AA1 with accession no. PX273401.1.

### 3.2 Phytochemical Analysis of *A. niger* Extract

The phytochemical analysis of the *A. niger* extract revealed a high concentration of bioactive substances. Due to their capacity to neutralize free radicals, flavonoid equivalent content was the most prevalent (496.5 µg/mL), suggesting strong antioxidant potential. Following at 260.7 µg/mL, the tannin‐equivalent content indicated potential antimicrobial and anti‐inflammatory properties through microbial inhibition and protein precipitation. The extract's antioxidant activity is further enhanced by phenolic compounds, which were measured at 214.9 µg/mL. Even so, Table 1 shows that alkaloid equivalent content was found at a lower concentration (26.4 µg/mL).

**Table 1:** Determination of total phenolics, alkaloid equivalent content, flavonoid equivalent content, and tannin equivalent content of *Aspergillus niger* extract.

Total phenolic (µg/mL)	214.93 ± 1.026
Tannin equivalent content (µg/mL)	260.76 ± 1.854
Flavonoid equivalent content (µg/mL)	496.53 ± 1.026
Alkaloid equivalent content (µg/mL)	26.45 ± 0.945

*Note:* Mean ± standard deviation (SD) of three replicates.

*A. niger* extract high flavonoid equivalent content indicated a potent ability to scavenge free radicals, providing substantial antioxidant protection. These substances can reduce cellular damage, halt oxidative chain reactions, and improve overall health resilience.

### 3.3 GC–MS Analysis of *A. niger* Extract

GC–MS analysis of the investigated extract identified 25 phytochemical compounds belonging predominantly to fatty acids, long‐chain hydrocarbons, fatty alcohols, sterols, triterpenoids, and aromatic compounds (Table 2 and Figure S1). Among the identified constituents, hexadecanoic acid was the most abundant compound, accounting for 7.7% of the total peak area, followed by docosene (6.4%), isochiapin B (5.5%), tris(2,4‐di‐*tert*‐butylphenyl) phosphate (5.3%), benzaldehyde,4‐nitro (4.1%), octadecenoic acid (4%), octadecanol (3.5%), behenic alcohol (3.5%), octadecane (2.8%), and octacosanol (2.6%). Other identified constituents included eicosane (2.4%), heptadecanoic acid (1.9%), vaccenic acid (1.6%), hexacosene (1.5%), lupeol (1.4%), pentadecanoic acid (0.9%), pentatriacontene (0.6%), dodecanoic acid (0.5%), erucic acid (0.5%), sitosterol (0.4%), diethyltoluamide (0.3%), hexadecane (0.2%), tetradecanoic acid (0.2%), tetradecanol (0.2%), and nonacosane (0.1%). The identified compounds have previously been reported to exhibit a broad spectrum of biological activities. Several constituents, including hexadecanoic acid, octadecenoic acid, hexadecane, isochiapin B, octadecane, heptadecanoic acid, docosene, erucic acid, sitosterol, and tris(2,4‐di‐*tert*‐butylphenyl) phosphate, possess antimicrobial activity.

**Table 2:** Characterization of *Aspergillus niger* extract by GC–MS.

No.	Compound	RT (min)	Peak area (%)
1	Benzaldehyde,4‐nitro	23.07	4.1
2	Tetradecanol	30.82	0.2
3	Diethyltoluamide	33.83	0.3
4	Dodecanoic acid	34.84	0.5
5	Hexadecane	36.19	0.2
6	Isochiapin B	38.44	5.5
7	Nonacosane	41.67	0.1
8	Tetradecanoic acid	41.83	0.2
9	Octadecanol	43.00	3.5
10	Octadecane	43.33	2.8
11	Pentadecanoic acid	44.31	0.9
12	Hexadecenoic acid	47.77	7.7
13	Docosene	49.51	6.4
14	Eicosane	49.82	2.4
15	Heptadecanoic acid	50.42	1.9
16	Vaccenic acid	53.87	1.6
17	Octadecenoic acid	54.00	4
18	Behenic alcohol	55.92	3.5
19	Pentatriacontene	56.64	0.6
20	Erucic acid	57.55	0.5
21	Octacosanol	62.66	2.6
22	Hexacosene	68.34	1.5
23	Sitosterol	83.46	0.4
24	Lupeol	84.85	1.4
25	Tris(2,4‐di‐*tert*‐butyl phenyl) phosphate	92.11	5.3

Abbreviations: GC–MS, gas chromatography–mass spectrometry; RT, room temperature.

### 3.4 Antimicrobial Activity

Table 3 and Figure 3 showed the antimicrobial activity of *A. niger* extract against clinically relevant microorganisms. *A. niger* extract showed a wide range of antimicrobial activity and inhibited the growth of all tested microbial strains in the current study. However, the susceptibility of the tested pathogens varied greatly by species. The highest sensitivity to *A. niger* extract was observed in *K. pneumoniae* with an inhibition zone diameter of 32 mm, then *S. aureus* (31.6 mm) and *B. cereus* (29.6 mm). These zones of inhibition indicated a significant antimicrobial potential of the *A. niger* extract against these tested pathogens. On the other hand, *E. coli* was moderately susceptible with an 18.3 mm inhibition zone. Similarly, *A. niger* extract was moderately active against *C. albicans* with an inhibition zone diameter of 17.6 mm.

**Table 3:** Antimicrobial activity of *Aspergillus niger* extract.

Microbial strain	Inhibition zone of *A*. *niger* extract (mm)	DMSO	Piperacillin/fluconazole
*Staphylococcus aureus*	31.6 ± 0.3	0	0
*Klebsiella pneumonia*	32 ± 0.5	0	0
*Bacillus cereus*	29.6 ± 0.3	0	0
*Escherichia coli*	18.3 ± 0.3	0	0
*Candida albicans* ATCC 10231	17.6 ± 0.3	0	16.6 ± 0.3

Abbreviation: DMSO, dimethyl sulfoxide.

**Figure 3:**
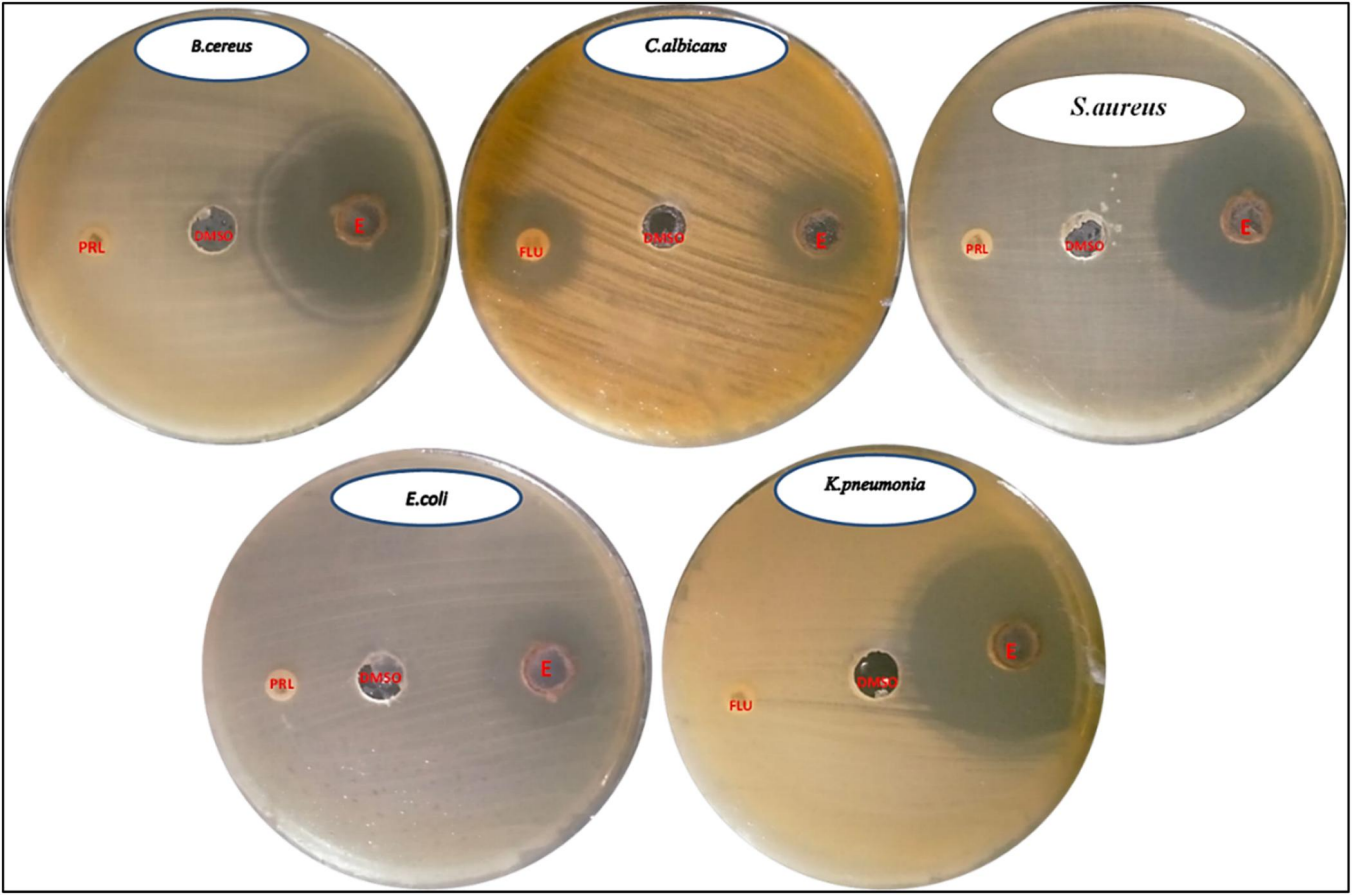
Antimicrobial activity of *Aspergillus niger* extract. E, *A. niger* extract; PRL, piperacillin.

The extract exhibited inhibitory activity against *S. aureus, B. cereus*, and *K. pneumoniae* confirmed with the MIC values of 62.5 μg/mL, as shown in Figure S2 and Table 4. The result showed a moderate inhibitory effect against *C. albicans* with an MIC value of 125 μg/mL. However, *E. coli* was the least susceptible microorganism, requiring a higher concentration of *A. niger* extract (250 μg/mL) to completely inhibit growth.

**Table 4:** MIC of *Aspergillus niger* extract against pathogenic bacterial and fungal isolates.

Microbial strain	MIC of *A. niger* extract (μg/mL)
*Staphylococcus aureus*	62.5
*Bacillus cereus*	62.5
*Klebsiella pneumonia*	62.5
*Escherichia coli*	250
*Candida albicans* (ATCC 10231)	125

Abbreviation: MIC, minimum inhibitory concentration.

The observed inhibition zones, especially against *K. pneumoniae, S. aureus*, and *B. cereus*, indicated the observed activity may be associated with the combined action of multiple fungal metabolites rather than a single bioactive constituent.

### 3.5 Antioxidant Activity

The free radical scavenging activity (FRSC) of *A. niger* extract was concentration dependent in both assays, as shown in Figures 4 and 5. The DPPH assay showed that the *A. niger* extract exhibited a high FRSC, with an IC_50_ of 167.6 μg/mL. Similarly, the ABTS assay confirmed the ability of *A. niger* extract to FRSC with an IC_50_ value of 137.8 µg/mL. DPPH radical is a relatively stable nitrogen‐centered free radical, usually dissolved in organic solvents such as methanol or ethanol; thus, the DPPH test is principally a measure of activity of lipophilic antioxidants and can underestimate contribution of hydrophilic compounds.

### 3.6 Effect of the *A. niger* Extract on Biofilm Biomass

Figures 3, 4, 6, and 5S showed the in vitro antibiofilm activity of *A. niger* extract against the tested pathogens. Results showed significant inhibition of biofilm formation across all microbial species tested in a concentration‐dependent way. *A. niger* extract was most effective against *C. albicans* with biofilm reduction between 55.1% ± 1% and 10.7% ± 1.6% at ½–⅛ MIC, respectively. On the other hand, *E. coli* showed a low sensitivity, with inhibition values decreasing from 26.9% at ½ MIC to 7.2% at ⅛ MIC.

**Figure 4:**
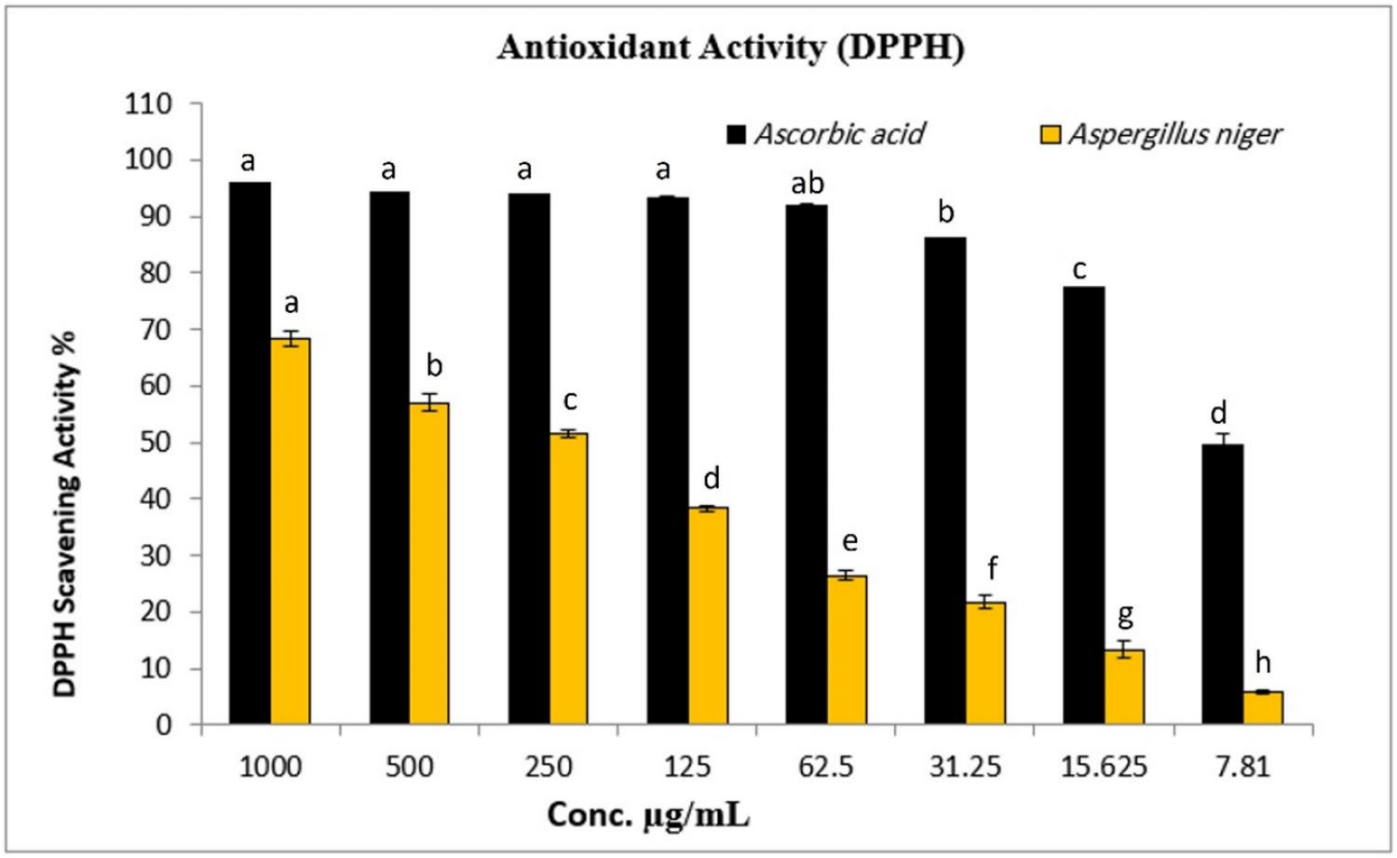
Antioxidant activity of *Aspergillus niger* extract using DPPH. Letters a, b, c, … mean significance power. DPPH, 1,1‐diphenyl‐2‐picrylhydrazyl.

**Figure 5:**
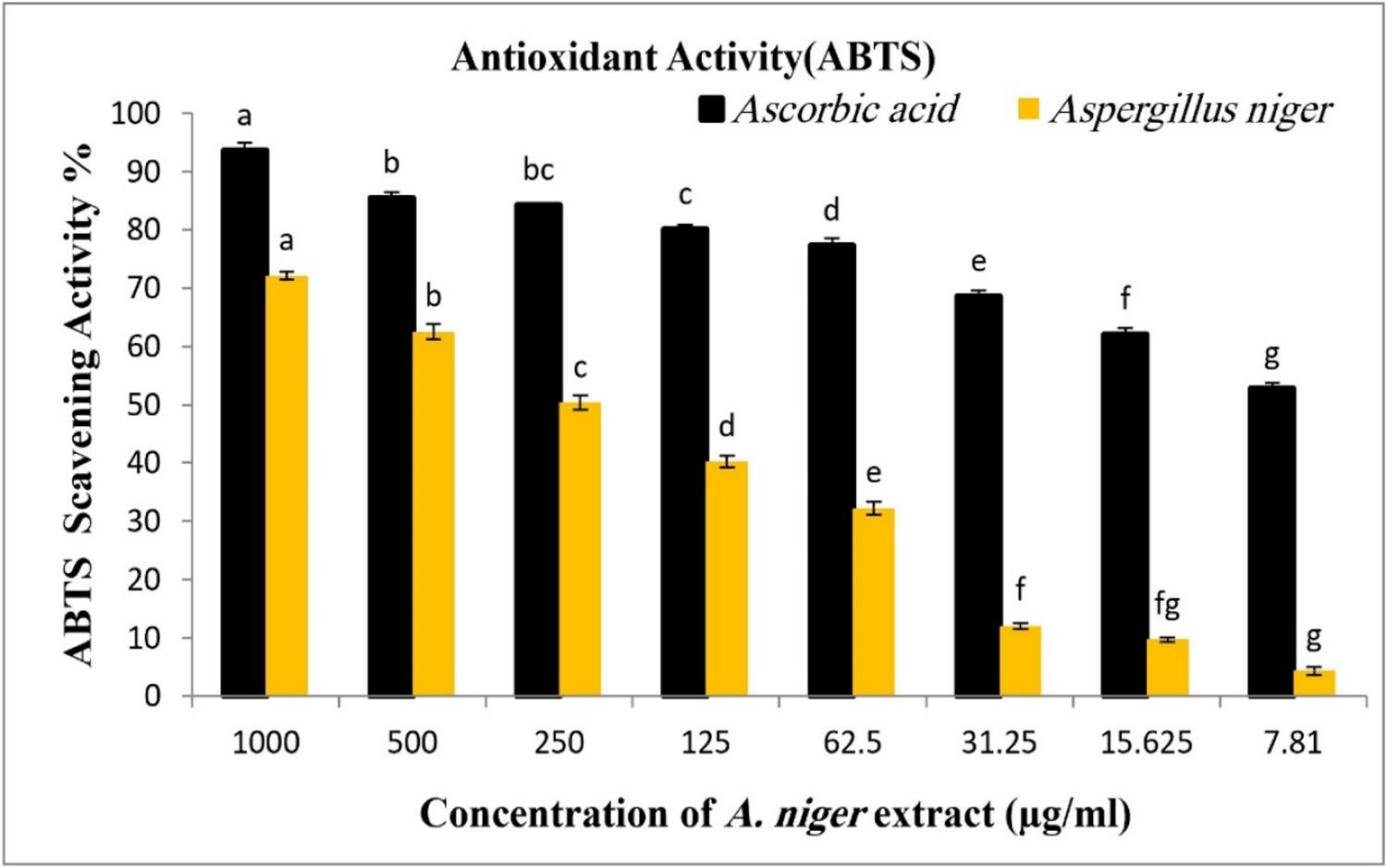
Antioxidant activity of *Aspergillus niger* extract using ABTS. Letters a, b, c, … mean significance power. ABTS, 2,2′‐azino‐bis‐3‐ethylbenzthiazoline‐6‐sulfonic acid.

**Figure 6:**
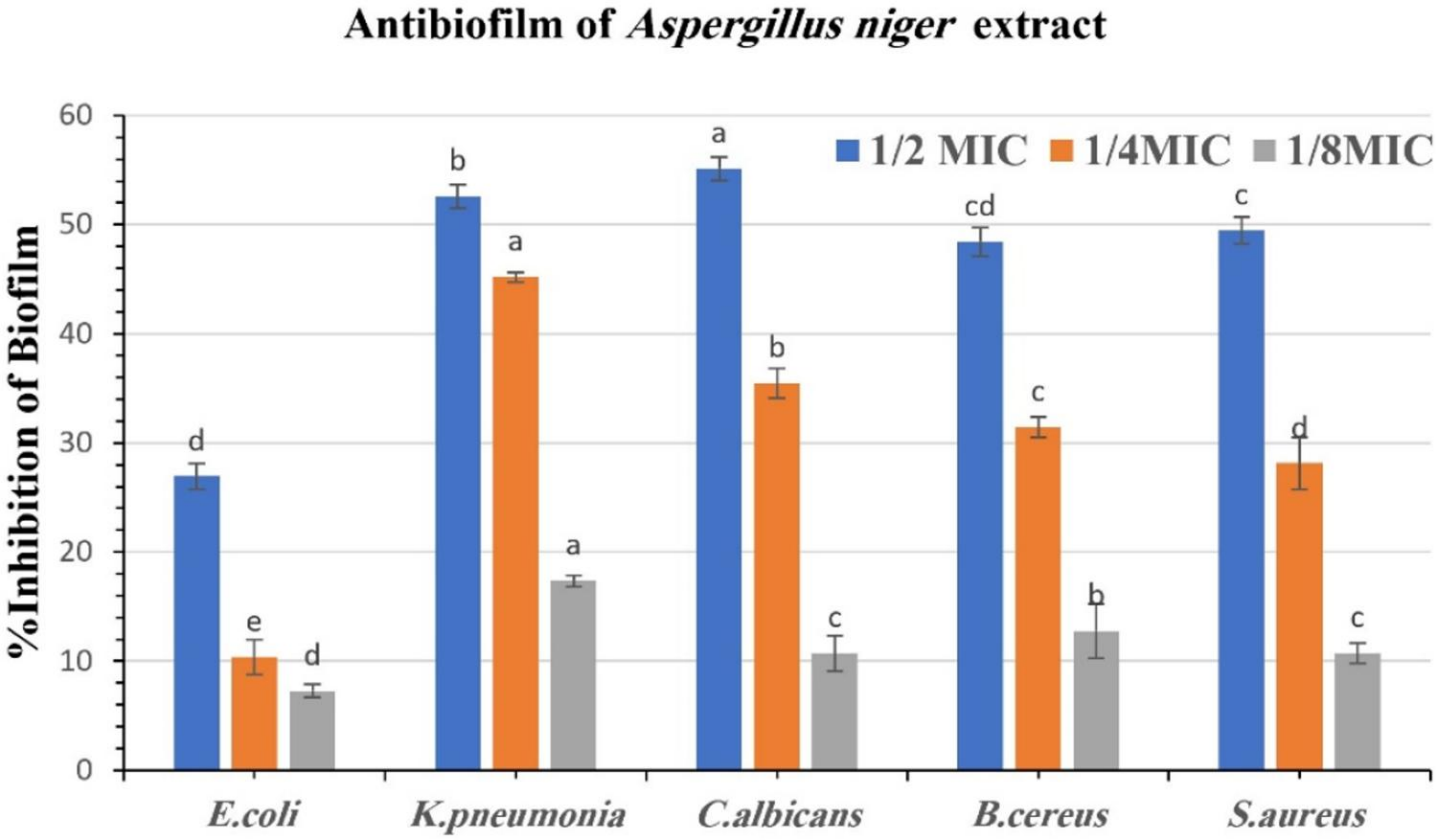
Effect of the *Aspergillus niger* extract on biofilm biomass of pathogens. Letters a, b, c, … mean significance power. MIC, minimum inhibitory concentration.

### 3.7 Cytotoxicity and Anticancer Activity

In the current study, the cytotoxicity of the *A. niger* extract was evaluated against Wi‐38 and Vero cell lines using the MTT assay, as shown in Figure 7A. The results demonstrated that the fungal extract exhibited relatively low cytotoxicity toward both normal cell lines in a concentration‐dependent manner, indicating its acceptable biocompatibility and safety profile. The IC_50_ values of the *A. niger* extract were 269.4 µg/mL for Wi‐38 cells and 396.8 µg/mL for Vero cells. In general, if the IC_50_ is ≥ 90 μg/mL. These findings suggest that the biosynthesized fungal extract possesses promising biological safety toward normal mammalian cells, which supports its potential application in biomedical and antimicrobial fields. The relatively high IC_50_ values further indicate selective activity with reduced toxicity to healthy cells, making the extract a promising candidate for future pharmaceutical and therapeutic applications.

**Figure 7:**
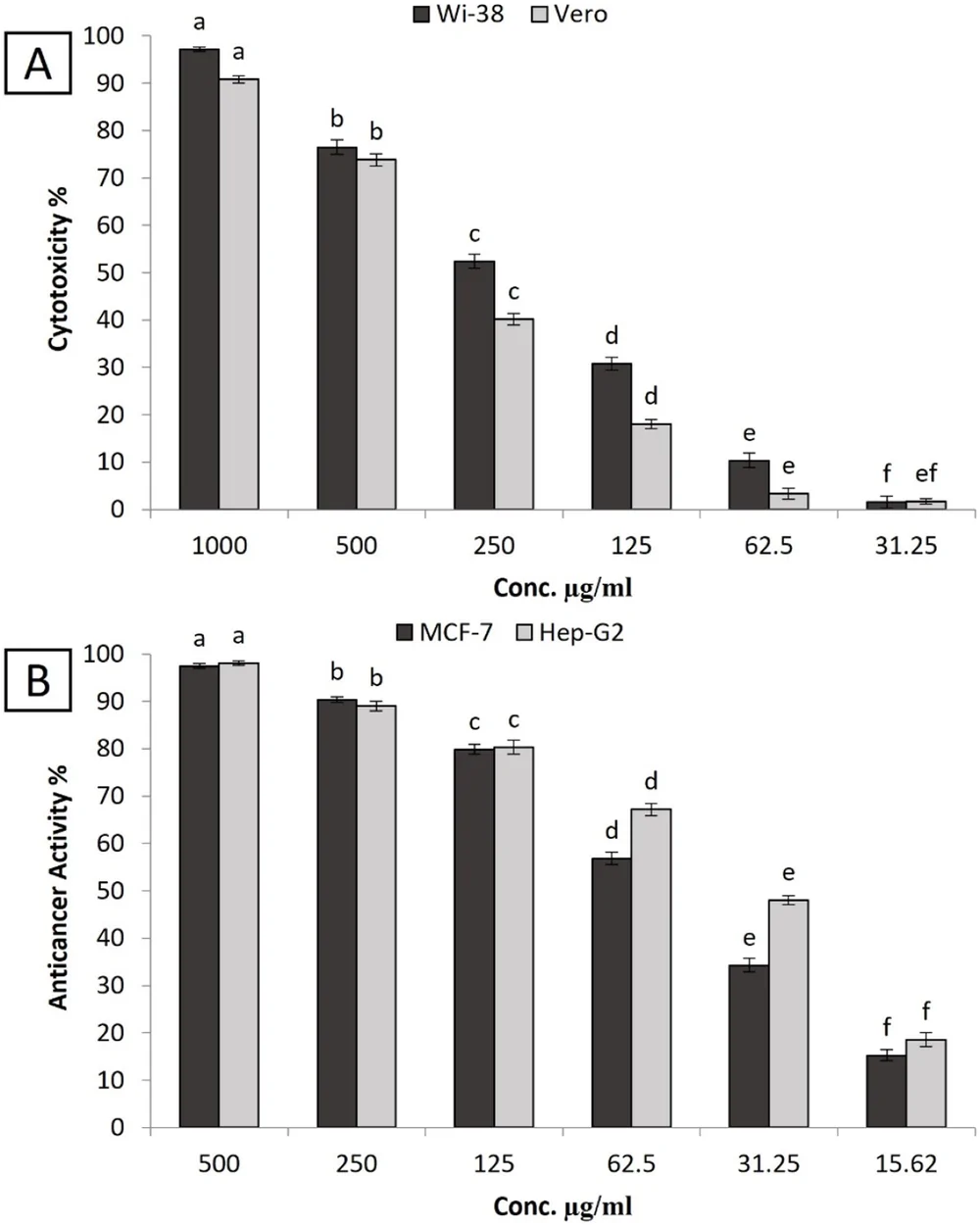
(A) Cytotoxicity of *Aspergillus niger* extract toward Wi‐38 and Vero normal cell lines and (B) anticancer activity of *A. niger* extract toward MCF‐7 and Hep‐G2 cancerous cell lines. Letters a, b, c, … mean significance power. MCF‐7, Michigan Cancer Foundation‐7.

Additionally, the extract of *A. niger* was assessed for its anticancer efficacy against MCF‐7 and Hep‐G2 malignant cell lines, as depicted in Figure 7B. The data obtained demonstrated a significant concentration‐dependent cytotoxic effect on both cancer cell lines. At the maximum evaluated concentration (500 µg/mL), the extract demonstrated significant anticancer efficacy, achieving inhibition rates of 97.4% and 98% against MCF‐7 and Hep‐G2 cells, respectively. The cytotoxic efficacy progressively diminished as the concentration decreased, reaching 15.2% for MCF‐7 and 18.5% for Hep‐G2 at 15.6 µg/mL. The determined IC50 values were 78.7 µg/mL for MCF‐7 cells and 29.5 µg/mL for Hep‐G2 cells, indicating that Hep‐G2 cells were more sensitive to the fungal extract than MCF‐7 cells. To verify the safety characteristics of these drugs, the SI was assessed. Employing the Wi‐38 cell line, the SI values for MCF‐7 and Hep‐G2 cells were recorded at 3.4 and 9.1, respectively, while the analogous SI values for the Vero cell line were 5 and 13.4. These findings indicate that both compounds exhibited acceptable selectivity toward cancer cells, as compounds with SI values greater than 2 are generally considered safe and selectively cytotoxic to malignant cells.

### 3.8 Molecular Docking

The selected ligands are the most abundant and therapeutically important bioactive compounds in the extract of *A. niger* for the selected endpoints (antimicrobial and anticancer activities). Hexadecenoic acid (7.7%) was selected for antimicrobial screening due to its high peak areas and known antibacterial activities. Conversely, isochiapin B and hexadecenoic acid were chosen for anticancer docking because the analysis directly links the compounds to the observed anticancer activity. Other compounds listed in Table 5 were discarded either because they were present in trace amounts (e.g., tetradecanol, 0.2%), which limited their potential therapeutic contribution, or because their main biological activities, such as insecticidal or anti‐inflammatory activities, were not directly related to the targeted antimicrobial or anticancer endpoints.

**Table 5:** Docking interaction data calculations of the test compounds and the control ligand (B47) within the active site of DNA gyrase subunit B (GyrB) (PDB ID 3G7B).

Compound	Ligand atom/moiety	Amino acid residue	Interaction type	Bond distance (Å)	Bond strength (kcal/mol)	Energy score (S) (kcal/mol)
Hexadecenoic acid	O 47	ARG144	H‐acceptor	2.92	−1.1	−7.2279
Control ligand (B47)	O 4	ARG144	H‐acceptor	3.14	−1.9	−6.2972
	O 4	ARG144	H‐acceptor	3.27	−1.8	

The docking protocol was validated by re‐docking of the co‐crystallized ligand (B47) into the active site of GyrB. The docking score of the control ligand was −6.2972 kcal/mol, and it had stabilizing hydrogen‐bond acceptor interactions with ARG144 at distances of 3.1 and 3.27 Å (Table 5 and Figure 8). Out of the compounds screened, hexadecenoic acid was found to be the best binding compound with a docking score of −7.2279 kcal/mol compared with the control ligand. The interaction of a hydrogen acceptor with ARG144 at a bond distance of 2.92 Å was mainly responsible for the stability of the ligand–protein complex. Despite forming fewer hydrogen bonds than the reference ligand, hexadecenoic acid demonstrated a more favorable overall docking score, suggesting that additional noncovalent interactions, such as hydrophobic and van der Waals interactions within the binding pocket, contribute to stabilizing the ligand–protein complex. The shorter hydrogen‐bond distance observed for hexadecenoic acid also indicates a stable interaction with the active‐site residue. Thus, these findings demonstrate that hexadecenoic acid possesses a strong binding affinity toward the catalytic pocket of GyrB and exhibits docking characteristics comparable to or better than those of the reference inhibitor B47.

**Figure 8:**
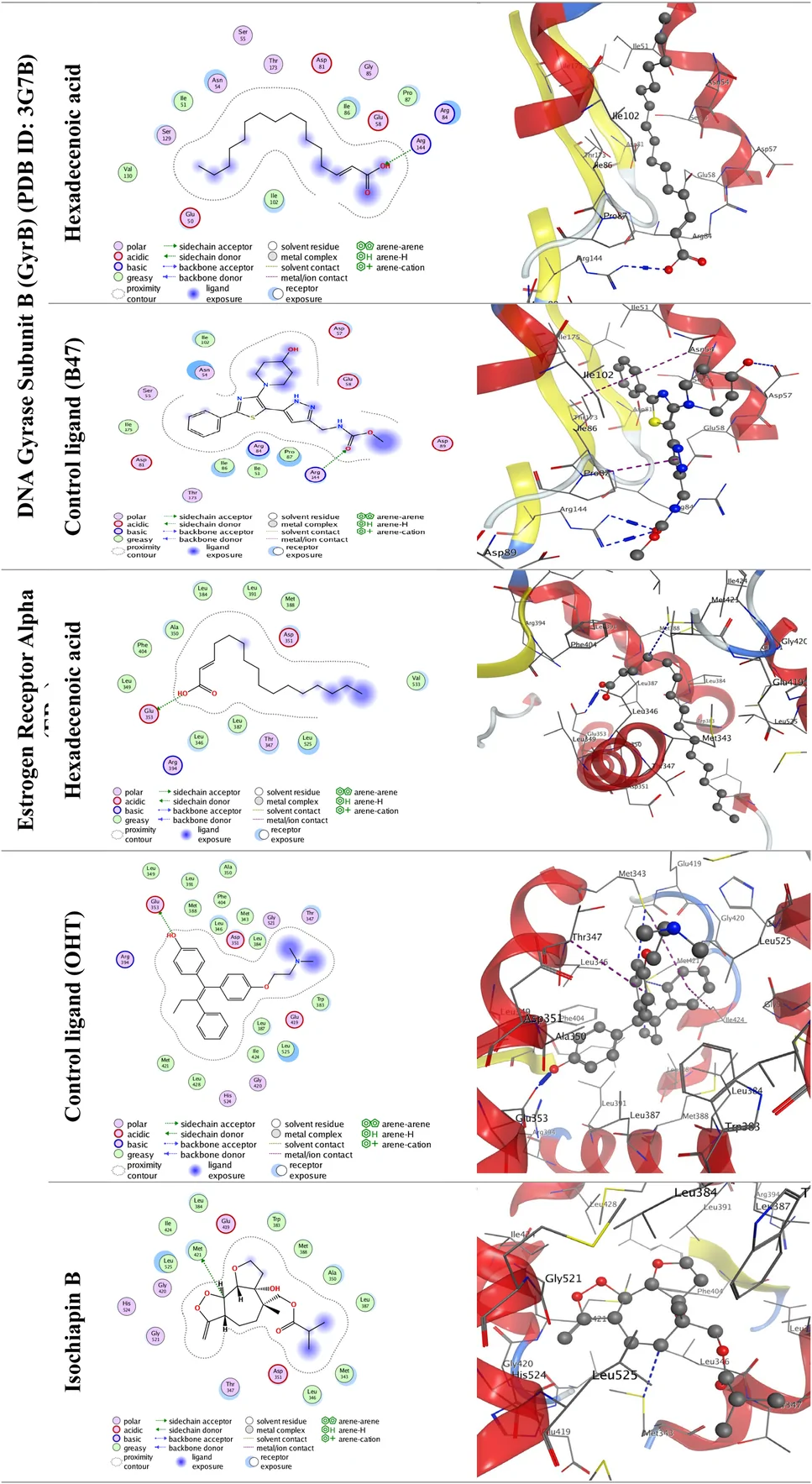
Two‐ and three‐dimensional molecular docking poses showing the binding modes and noncovalent interactions of the test compounds and control ligands (B47 and OHT) within the active sites of GyrB (PDB ID 3G7B) and ERα (PDB ID 3ERT). ERα, estrogen receptor alpha; GyrB, gyrase subunit B; OHT, 4‐hydroxytamoxifen; PDB, Protein Data Bank.

The ERα docking protocol was validated by re‐docking the co‐crystallized OHT ligand, which showed a docking score of −6.2501 kcal/mol and an H‐bond donor interaction with GLU353 (2.65 Å). Among the tested compounds, prostaglandin A1‐biotin exhibited the highest ERα binding affinity (−9.6557 kcal/mol), forming H‐bond interactions with LEU346 (3.13 Å), CYS530 (3.22 Å), TYR526 (2.83 Å), and THR347 (2.70 Å) (Table 6 and Figure 8). Hexadecenoic acid also showed a stronger binding compared with the control ligand, with a dock score of −6.3531 kcal/mol. The key hydrogen donor interaction of the molecule with GLU353 was mimicked at a bond distance of 2.81 Å, and the molecule showed a similar binding mode as that of the reference ligand. The docking score of isochiapin B was −6.2456 kcal/mol and is comparable with the score of the control ligand. The stability of its complex was attributed to a hydrogen donor interaction with MET421 at a bond distance of 3.68 Å (Table 6 and Figure 8). Thus, both test compounds demonstrated favorable binding within the ERα active site. Among them, hexadecenoic acid displayed the most favorable docking score and the strongest hydrogen‐bond interaction, outperforming the reference ligand, whereas isochiapin B exhibited a comparable docking score but formed a weaker interaction with a different amino acid residue. These results suggest that hexadecenoic acid possesses the highest predicted binding affinity toward ERα among the tested compounds.

**Table 6:** Docking interactions of the test compounds and control ligand (OHT) within the ERα active site (PDB ID 3ERT).

Compound	Ligand atom/moiety	Amino acid residue	Interaction type	Bond distance (Å)	Bond strength (kcal/mol)	Energy score (S) (kcal/mol)
Hexadecenoic acid	O 47	GLU353	H‐donor	2.81	−8	−6.3531
Control ligand (OHT)	O 11	GLU353	H‐donor	2.65	−5	−6.2501
Isochiapin B	C 18	MET421	H‐donor	3.68	−0.9	−6.2456

Abbreviations: ERα, estrogen receptor alpha; OHT, 4‐hydroxytamoxifen; PDB, Protein Data Bank.

## 4 Discussion

In this study, five endophytic fungal isolates, AA1, AA2, AA3, AA4, and AA5, were successfully isolated from *D. tortuosa* and screened for their antimicrobial potential. Among the tested isolates, AA1 showed the highest antimicrobial activity and was selected for further investigation and identified as *A. niger*. *A. niger* is one of the most commonly reported fungal endophytes isolated from various medicinal, agricultural, and wild plants. In many studies, endophytic *A. niger* has demonstrated the capacity to produce valuable secondary metabolites, enzymes, and antimicrobial compounds that enhance stress resistance and protect plants against phytopathogenic microorganisms (Gao et al. 2025; Aziz et al. 2021). The presence of *A. niger*, previously reported as an endophyte in *Acacia arabica*, Mangrove, *Trifolium alexandrinum* plants in recent studies (Liu et al. 2011; Attia 2025b). The host plant is an important ecological and biochemical environment that may affect the growth of endophytes, nutrient availability, and the expression of biosynthetic pathways, thereby influencing the qualitative and quantitative composition of endophytic fungal secondary metabolites (Li et al. 2022). The isolation of *A. niger* from the medicinal and desert‐adapted plant *D. tortuosa* provides another host‐endophyte system for exploring the chemical and biological diversity of this fungus. In this sense, the antimicrobial, antibiofilm, antioxidant and cytotoxic activities obtained in the present study, as well as the unique GC–MS profile obtained from the EACE, suggest that the association *D. tortuosa A. niger* could be a valuable source of bioactive fungal metabolites (Zhang and White 2021).

Phytochemical analysis of *A. niger* extract revealed the presence of a broad spectrum of bioactive constituents, particularly flavonoids, phenolics, tannins, and alkaloids, indicating its potential antioxidant, antimicrobial, and health‐promoting properties. Flavonoids are significant secondary metabolites well known for their antibacterial, anti‐inflammatory, and antioxidant properties (Panche et al. 2016). Furthermore, significant amounts of tannins were discovered in *A. niger* extract. These polyphenolic compounds have the potential to be used therapeutically to treat infections and disorders related to inflammation because of their well‐known capacity to suppress microbial growth and produce astringent effects (Scalbert 1991). By shielding cells from free radicals and reducing disease risk, flavonoids have a major positive impact on health. By affecting angiogenesis and key pathways such as PI3K/Akt and MAPK, they help prevent cancer. Their effects on lung, liver, colon, and breast cancers are particularly noteworthy (Pyo et al. 2024). Phenolic compounds primarily exert antioxidant effects by donating hydrogen atoms or electrons to neutralize reactive oxygen species and prevent cellular oxidative damage (Attia 2025b). Alkaloids are diverse nitrogen‐containing compounds with well‐established pharmacological activities. They have traditionally been used to treat various conditions, while their potent bioactivities have also contributed significantly to modern drug development. Several alkaloids, including morphine, codeine, quinine, and ephedrine, remain important therapeutic agents (Gutiérrez‐Grijalva 2020).

The GC–MS analysis of the ethyl acetate extract of *A. niger* indicated the tentative identification of 25 metabolites. Major constituents were hexadecenoic acid (7.7%), docosene (6.4%), benzaldehyde,4‐nitro (4.13%), octadecenoic acid (4.0%), octadecanol (3.52%), and behenic alcohol (3.5%). Some of these compounds have been previously reported to possess antimicrobial and antioxidant activities that may contribute to the observed biological activities of the crude extract. Hexadecenoic acid, a fungal secondary metabolite with antimicrobial activity against pathogens (Y. M. Ahmed et al. 2026; Abdelaziz et al. 2022). Long‐chain fatty acids and related alcohols, such as erucic acid, octadecanol, octadecanoic acid, and octacosanol, have also been shown to exhibit membrane‐disrupting, antimicrobial, and/or antioxidant properties (Ma et al. 2026). This provides a reasonable chemical basis for the antimicrobial and antioxidant activities of *A. niger* extract. Thus, the metabolites found together could have an additive or synergistic effect on the extract's overall activity. The biological assays were carried out with the crude extract, so the detected metabolites may act alone or in combination, and their specific contribution should be confirmed by isolation, purification, and bioactivity‐guided evaluation.

The strong antibacterial activity against Gram‐positive *S. aureus* and *B. cereus* may be attributed to their simpler cell envelopes, which lack the outer lipopolysaccharide membrane found in Gram‐negative bacteria, thereby facilitating antimicrobial penetration through the peptidoglycan layer (Xu et al. 2026). Moreover, phytochemicals such as phenolics, flavonoids, tannins, terpenoids, and alkaloids can exert antimicrobial effects through membrane disruption, protein denaturation, inhibition of nucleic acid synthesis, and interference with essential metabolic enzymes (Duffey and Stout 1996). Interestingly, *A. niger* extract also showed significant activity against *K. pneumoniae*, a Gram‐negative pathogen, which is usually more resistant to antimicrobial agents due to the presence of an outer membrane that serves as an effective permeability barrier. The exceptionally large inhibition zone (32 mm) indicates that certain constituents of the *A. niger* extract can overcome this barrier and exert potent antibacterial effects. This activity may be due to low‐molecular‐weight phenolic compounds or lipophilic metabolites that interact with membrane lipids, increasing membrane permeability and the leakage of important intracellular constituents (Helander et al. 1998). On the other hand, the comparatively lower susceptibility of *E. coli* may be related to intrinsic characteristics commonly associated with Gram‐negative bacteria, including reduced membrane permeability and active efflux systems.

The moderate antifungal activity against *C. albicans* indicates that *A. niger* extract may also interfere with the cellular processes of fungi. The antifungal effect might be due to the interaction of phytochemicals with fungal membranes containing Ergosterol, resulting in changes in membrane integrity, increased permeability, and disruption of cellular homeostasis. Phenolic compounds and terpenoids have been reported extensively to inhibit fungal growth by inducing oxidative stress, mitochondrial dysfunction, and impairment of cell wall biosynthesis. Phenolic compounds known to destabilize microbial membranes and inhibit enzyme systems, whilst tannins may precipitate microbial proteins and interfere with nutrient uptake (Beckman and Roberts 1995). The combined effect of secondary metabolites is often stronger than the effect of individual compounds on antimicrobial activity.

The *A. niger* extract exhibited strong free‐radical scavenging activity, with IC_50_ values of 167.6 and 137.8 μg/mL for DPPH and ABTS, respectively. The higher ABTS activity may indicate a greater abundance of polar antioxidants, as ABTS radicals react with both hydrophilic and lipophilic compounds (Çelik et al. 2010). Another explanation refers to the reaction kinetics and the steric accessibility of the radicals. The DPPH radical is sterically hindered and relatively bulky, which may limit its interactions with certain antioxidant molecules, particularly high‐molecular‐weight phenolics and tannins. By contrast, ABTS radicals are more accessible and react more rapidly with a broader range of antioxidant compounds (Foti 2015). Therefore, *A. niger* extract rich in phenolic acids, flavonoids, tannins, and other polyphenolic metabolites generally demonstrates higher antioxidant activity in the ABTS assay compared with the DPPH assay. Therefore, the increased ABTS FRSC of *A. niger* extract may be associated with its phytochemical composition. The phytochemical screening demonstrated high concentrations of total phenolics, flavonoid‐equivalent content, and tannin‐equivalent content, which could account for a large part of the observed antioxidant capacity. The phenolic hydroxyl groups can readily donate electrons or hydrogen atoms to stabilize ABTS radicals, and flavonoid and tannin equivalent contents can further enhance FRSC through resonance stabilization and metal‐chelating mechanisms. Many studies have further supported the role of polyphenols as major contributors to antioxidant potential by reporting a significant positive correlation between TPC and ABTS radical‐scavenging activity (Babbar et al. 2011).

The antioxidant activity of *A. niger* extract may be attributed to phenolics, flavonoids, and tannins identified by GC–MS, which contribute through electron/hydrogen donation, radical scavenging, metal chelation, and reducing activity (Rice‐Evans et al. 1997; Van Acker et al. 1996). The extract also reduced biofilm biomass in a concentration‐dependent manner, with the strongest effect against *C. albicans*. Fatty acids and derivatives may contribute by disrupting microbial membranes and electron transport, while unsaturated fatty acids such as vaccenic acid can interfere with QS and biofilm development (Hoang 2000). This provides a direct mechanistic explanation for the strong concentration‐dependent antibiofilm activities observed in the present study, especially against *C. albicans*. Importantly, octacosanol has also been associated with antioxidant activity, which may indirectly contribute to antibiofilm effects by modulating signaling pathways related to oxidative stress that are involved in microbial aggregation (Getahun et al. 2023). Prostaglandin derivatives that indicated in *A. niger* extract are also known to modulate redox signaling and cellular communication which may further perturb microbial biofilm regulation (Fourie 2016). The antioxidant activity observed in the DPPH and ABTS assays could be mechanistically correlated with GC–MS constituents such as hexadecanoic acid, octadecanol, octacosanol, and phenolic‐like aromatic compounds. The metabolites could scavenge ROS and relieve oxidative stress. It is becoming clear that oxidative stress controls biofilm formation and microbial stress adaptation (Gambino and Cappitelli 2016).

Assessment of the cytotoxicity and biosafety of newly developed compounds or natural extracts is an essential step to ensure their safety toward normal human cells before biomedical or pharmaceutical applications. These assessments help determine the therapeutic potential, safe dosage range, and possible side effects of the tested materials, thereby supporting their future clinical and industrial use (Mosdam 1983; Araújo 2016). In general, if the IC_50_ is ≥ 90 μg/mL, the substance is classified as noncytotoxic (Ioset 2009). These findings suggest that the biosynthesized fungal extract exhibits promising biological properties and relatively low cytotoxicity toward the tested normal cell lines under the present in vitro conditions, supporting its potential applications in biomedical and antimicrobial fields. The relatively high IC_50_ values further indicate selective activity with reduced toxicity to healthy cells, making the extract a promising candidate for future pharmaceutical and therapeutic applications. Our findings suggest that the *A. niger* extract exhibits promising antiproliferative activity, particularly against liver cancer cells, and may contain potent bioactive metabolites that inhibit cancer cell growth. The higher sensitivity of Hep‐G2 cells may be attributed to differences in cellular metabolism, membrane permeability, and oxidative stress response between the tested cancer cell lines. The extract exhibited concentration‐dependent cytotoxicity against HepG2 and MCF‐7 cells, potentially due to secondary metabolites such as alkaloids, polyketides, terpenoids, peptides, and phenolics that can induce apoptosis, ROS generation, mitochondrial dysfunction, and cell‐cycle arrest (Jangid et al. 2024; Yu et al. 2021). Consistently, *A. niger* metabolites have demonstrated anticancer activity, with *A. niger* AW17 extract showing strong activity against HepG2 (IC_50_ = 5.22 µg/mL) and lower toxicity toward normal cells (Zina et al. 2025).

In silico binding affinity showed a strong correlation with the putative antimicrobial and anticancer potential of the bioactive metabolites identified from *A. niger*. Molecular docking analysis showed that hexadecenoic acid has favorable predicted interactions with DNA GyrB and ERα via ARG144 and GLU353, respectively. These results may provide a molecular explanation for the antimicrobial and anticancer activities observed in the *A. niger* extract, based on the reported bioactivities of fatty acid–related metabolites (N. Ahmed et al. 2023). The observed residue‐specific interactions with ARG144 in GyrB and GLU353 in ERα indicate that hexadecenoic acid is accommodated within the biologically relevant active sites of both target proteins, suggesting its potential to inhibit bacterial growth and interfere with cancer‐related signaling pathways.

It is important to understand the correlation between the in vitro biological activities and the molecular docking results. The present study evaluated the EACE of *A. niger* for antimicrobial, antioxidant, and anticancer activities, as it is a complex mixture of multiple secondary metabolites. Therefore, based on the in vitro assays, the biological effects observed cannot be attributed to a single compound. In contrast, the molecular docking analysis was performed on selected individual compounds present in the extract, such as hexadecenoic acid and isochiapin B, to study their potential interactions with the selected molecular targets.

The strong binding affinities and specific interactions identified by molecular docking provide in silico mechanistic support for the possible contribution of these metabolites to the biological activities observed in the crude extract. However, the docking results should not be considered as direct evidence for the sole responsibility of these individual compounds for the observed activity, as the crude extract contains multiple constituents that could act independently, additively, synergistically, or antagonistically. Thus, the docking results can be considered a complementary tool to the in vitro results and can be useful for identifying potentially bioactive constituents and their possible molecular targets. To definitively attribute the observed biological activities to hexadecenoic acid, isochiapin B, or other individual metabolites, it would be necessary to isolate and purify these compounds and test them, ideally with appropriate mechanistic validation.

## 5 Conclusion

In conclusion, the endophytic fungus *A. niger* AA1 represents a promising source of biologically active metabolites with potential biomedical applications. Integrated morphological, molecular, phytochemical, and GC–MS analyses revealed a diverse metabolite profile containing phenolics, flavonoids, tannins, alkaloids, and other bioactive constituents. The fungal extract exhibited broad‐spectrum antimicrobial and antibiofilm activities against both Gram‐positive and Gram‐negative bacteria, including *C. albicans*, along with considerable antioxidant activity in DPPH and ABTS assays. Moreover, the extract showed low cytotoxicity toward normal mammalian cells while exhibiting selective antiproliferative effects against Hep‐G2 and MCF‐7 cells, with favorable selectivity indices. Molecular docking further indicated strong interactions of selected fungal metabolites with GyrB and ERα, supporting their potential antimicrobial and anticancer roles. Therefore, these findings highlight *A. niger* AA1 as a valuable natural reservoir of multifunctional metabolites and warrant further mechanistic and in vivo investigations toward their development as potential antimicrobial and anticancer agents.

## Author Contributions

**Samy Selim:** methodology, conceptualization, writing – review and editing, writing – original draft. **Amr H. Hashem:** conceptualization, writing – review and editing, writing – original draft, methodology, formal analysis, data curation. **Amer Morsy Abdelaziz:** conceptualization, writing – review and editing, writing – original draft, investigation, visualization. **Mohamed H. Sharaf:** conceptualization, writing – review and editing, writing – original draft, formal analysis, methodology. **Samiah Hamad Al‐Mijalli:** writing – review and editing, writing – original draft, funding acquisition, methodology. **Mohammed Aladhadh:** methodology, writing – review and editing. **Emad M. Abdallah:** visualization, investigation, software. **Mohammed H. Alruhaili:** methodology, writing – review and editing. **Hattan S. Gattan:** methodology, writing – review and editing. **Alaa A. Kashmiry:** methodology, writing – review and editing. **Mohammed Aufy:** writing – review and editing, data curation, methodology, formal analysis.

## Ethics Statement

The authors have nothing to report.

## Conflicts of Interest

None declared.

## Supporting information


Supporting File 1

## Data Availability

The data that support the findings of this study are available on request from the corresponding author. The data are not publicly available due to privacy or ethical restrictions.
