# Supplementary material for: Aspergillus niger as a New Endophytic Fungus From Deverra tortuosa: Phytochemical Profile, Molecular Docking, Antimicrobial, Antibiofilm, Antioxidant, and Anticancer Activities
Source: Microbiologyopen. 2026 Oct 7;15(5):e70430. doi: 10.1002/mbo3.70430 (PMC13646716; doi:10.1002/mbo3.70430)
Supplement: Supplementary file 1 [file MBO3-15-e70430-s001.docx]

**Figure S1:** GC-MS analysis of *A. niger* extract

**Figure S2: MIC of *A. niger* extract on tested microbial isolates.**

**Figure S3:** Crystal violet microtiter-plate assay illustrating the concentration-dependent (1/2 MIC: 1/128 MIC) inhibition of biofilm formation by the ethyl acetate extract of *Aspergillus niger* against the tested pathogens. The blue color represents bacterial biofilm formation, while clear or weakly stained wells indicate inhibition or reduction of biofilm biomass after treatment with increasing concentrations of the fungal extract.

**Figure S4:** Scanning electron microscopy (SEM) images illustrating the biofilm formation by *C. albicans*, the most sensitive for *Aspergillus niger* extract as biofilm inhibitor. SEM was operated at an accelerating voltage of 30 kV, with a working distance of 13.1 mm and a magnification of 1200×.

**Figure S5:** Scanning electron microscopy (SEM) images illustrating the anti biofilm formation of *Aspergillus niger* extract at ½ MIC against *C. albicans*. SEM was operated at an accelerating voltage of 30 kV, with a working distance of 12.4 mm and a magnification of 1200×.
